# Enhanced Microglia Activation and Glioma Tumor Progression by Inflammagen Priming in Mice with Tumor Necrosis Factor Receptor Type 2 Deficiency

**DOI:** 10.3390/life11090961

**Published:** 2021-09-14

**Authors:** Chih-Kai Liao, Kuan-Min Fang, Hui-Ting Huang, Wen-Ruei Chang, Chao-Chi Chuang, Shun-Fen Tzeng

**Affiliations:** 1 Institute of Life Sciences, College of Bioscience and Biotechnology, National Cheng Kung University, Tainan 70101, Taiwan; ckliao37@csmu.edu.tw (C.-K.L.); aleskuan@gmail.com (K.-M.F.); 10904022@gs.ncku.edu.tw (H.-T.H.); r9052000@hotmail.com (W.-R.C.); chaung.gi@gmail.com (C.-C.C.); 2 Department of Anatomy, Faculty of Medicine, Chung Shang Medical University, Taichung 40241, Taiwan; 3 Department of Medical Education, Faculty of Medicine, Chung Shang Medical University, Taichung 40241, Taiwan

**Keywords:** glioma, tumor necrosis factor receptor type 2, microglia, inflammation, lipopolysaccharide

## Abstract

Despite the fact that accumulation of microglia, the resident macrophages of the central nervous system (CNS) are the main feature of glioblastoma, the role of microglia in the progression of glioma is still arguable. Based on the correlation of inflammation with tumor progression, in this study, we attempt to determine if peripheral inflammation aggravates glioma expansion and the activation of microglia associated with the tumor. Experimental animals were administered intraperitoneally by inflammagen lipopolysaccharide (LPS) for 7 days (LPS priming) before intracerebral implantation of glioma cells. Moreover, a reduced level of tumor necrosis factor receptor type 2 (TNFR2) that is restricted to immune cells, neurons, and microglia has been found in patients with glioblastoma through the clinic analysis of monocyte receptor expression. Thus, in addition to wildtype (WT) mice, heterogeneous TNFR2 gene deficiency (*TNFR2*^+/−^) mice and homogeneous TNFR2 gene knockout (*TNFR2*^−/−^) mice were used in this study. The results show that peripheral challenge by LPS, Iba1^+^- or CD11b^+^-microglia increase in numbers in the cortex and hippocampus of *TNFR2*^−/−^ mice, when compared to WT or *TNFR2*^+/−^ mice. We further conducted the intracerebral implantation of rodent glioma cells into the animals and found that the volumes of tumors formed by rat C6 glioma cells or mouse GL261 glioma cells were significantly larger in the cortex of *TNFR2*^−/−^ mice when compared to that measured in LPS-primed WT or LPS-primed *TNFR2^+/−^* mice. Ki67^+^-cells were exclusively clustered in the tumor of LPS-primed *TNFR2*^−/−^ mice. Microglia were also extensively accumulated in the tumor formed in LPS-primed *TNFR2*^−/−^ mice. Accordingly, our findings demonstrate that aggravation of microglia activation by peripheral inflammatory challenge and a loss of TNFR2 function might lead to the promotion of glioma growth.

## 1. Introduction

Malignant glioma cells are the majority of primary brain tumors, which are ranked as the third greatest cause of cancer death among young and middle-aged adults [1,2]. Astrocytoma WHO grade IV or Glioblastoma are the most common and aggressive form of the tumors in the central nervous system (CNS) and account for over 50% of primary CNS tumors [1,3]. Based on the development of current therapies, the survival time of most patients with glioblastoma has been significantly improved up to 21 months on average [4,5]. Yet, this life expectancy is considerably short because the efficacy remains limited for high-grade glioma.

Resident macrophages in the CNS named microglia are differentiated from primitive myeloid cells that enter into the CNS during embryonic development [6]. Microglia display a ramified shape with a small cell soma and fine cell processes in healthy CNS; moreover, these cells, like other glia (i.e., astrocytes and oligodendrocytes), are responsible for the maintenance of microenvironmental homeostasis, such axonal pruning and synaptic modeling [7]. Alternatively, microglia rapidly respond to CNS injury alongside transformation of hypertrophic or amoeboid-like forms [8] and trigger neuroinflammation [7]. Microglia, macrophages, and infiltrating immune cells account for 30–50% of the total cell populations in glioma [9]. The tumor-associated microglia and macrophages are associated with the grade of gliomas [1,4,10]. Studies have indicated that microglia and macrophages serve as component cells in the microenvironment to expand glioma growth [4,11,12,13,14]. Microglia and macrophages that can promote tumor growth have also been reported to exist in gliomas [15]. Moreover, infiltrating microglia/macrophages are thought to serve as the molecule-producing cell sources that are needed to maintain an immunosuppressive glioma-associated milieu [1,4]. Despite that, according to proinflammatory M1 and immune suppressive M2 gene expression, microglia and macrophages are classified to be either anti-tumoral or pro-tumorigenic [16], the existence of M1 and M2 microglia/macrophage subtypes has been argued [17]. Indeed, in addition, the presence of microglia and macrophages with M2 markers predicts a poor survival factor in gliomas. Macrophages with M1 gene signatures, which are considered to be anti-tumoral, are also negatively correlated with glioma patient survival [9]. Nevertheless, the tumor-supporting role of reactive microglia in the inflamed brain is less addressed.

In this study, we aimed to examine the role of the activated microglia in glioma growth and chronic inflammation links to tumor initiation and progression [18,19]. Peripheral administration with inflammagen lipopolysaccharide (LPS) induces microglial activation and increases proinflammatory factors from hours to months [20,21]. In addition, tumor necrosis factor type II (TNFR2) expression is restricted to immune cells, endothelial cells, neurons, and microglia [22]. Clinical evidence has shown that TNFR2 expression is lower in the cell surface of monocytes isolated from glioblastoma patients when compared to those from normal patients or patients with non-glioma metastases [23]. Thus, to determine if glioma growth is enhanced in the inflamed brain, we conducted microglia priming by peripheral injection of LPS in TNFR2 deficient mice before the intracerebral implantation of xenogeneic or syngeneic glioma cells into the brain. The study showed that the activation of microglia in the cortical and hippocampal regions was significantly increased in TNFR2-deficient mice with peripheral LPS injection. Furthermore, aggressive glioma expansion and abundant accumulation of microglia at the tumor site were induced in TNFR2-deficient mice receiving peripheral LPS challenge. The study demonstrates that reactive microglia/macrophages promote glioma expansion, suggesting the activation of microglia/macrophages as a therapeutic target for anti-glioma.

## 2. Materials and Methods

Cell culture. The immortalized murine microglial cell line, BV2, was grown in Dulbecco’s Modified Eagle’s medium (DMEM)/F-12 (Gibco, Grand Island, NY, USA) containing 5% heat-inactivated fetal bovine serum (FBS; Hyclone, Logan, UT, USA), 50 U/mL penicillin and 50 mg/mL streptomycin (P/S; Invitrogen, Carlsbad, CA, USA). The C6 rat glioma cell line was from the National Health Research Institute Cell Bank (Zhunan, Taiwan). The mouse glioma cell line GL261 was provided by Dr. Kuo-Chen Wei (Department of Neurosurgery, Chang-Gung Memorial Hospital, Linkou, Taiwan). Glioma cells were grown in culture flasks in DMEM with 10% FBS and P/S. For cell implantation into C57BL/6 mice, C6 and GL261 cells were detached from a culture flask using 0.0025% trypsin/EDTA. After centrifugation, the cell pellet was re-suspended in sterilized PBS to a final dilution of 1 × 10^5^ cells/μL.

Lentivirus-mediated shRNA targeting *TNFR2***.** The shRNA-mediated knockdown of *TNFR2* was performed using shRNA lentiviral particles (Biosettia Inc., San Diego, CA, USA). The lentivirus vector constructs used in this study included the following: pLV-mU6-[sh-scramble]EF1a-GFP-puromycin (sh-ctrl), pLV-mU6-[sh-*TNFR2*-188]-EF1a-GFP-puromycin (sh-188), and pLV-mU6-[sh-*TNFR2*-220]-EF1a-GFP-puromycin (sh-220). BV2 cells at a density of 5 × 10^4^ cells per dish were seeded onto 35 mm dishes. After 1 day of seeding, 200 μL of lentiviral particles in 2 mL DMEM/F12 medium containing 10% FBS were added to the cultures as followed by the protocol described previously [24]. The cultures were then incubated for 24 h in 5% CO_2_ at 37 °C. The cells that were successfully infected by lentiviral particles were selected using 3 μg/mL puromycin in the presence of 10% FBS for 48 h. The transfectants were then harvested and subjected to QPCR to determine the efficiency of lentiviral particles used for the suppression of TNFR2 gene expression (Figure S1A). Since TNFR2 mRNA expression in BV2 cells was suppressed efficiently by sh-188 and sh-220, the cells transduced by sh-188 (*TNFR2*KD-BV2) were used for the in vitro study. The BV2 culture transduced by sh-ctrl (Scramble-BV2) was a control group.

C6 glioma-BV2 cell co-culture. C6 cells were replated at the density of 3 × 10^4^ cells/well onto the coverslip in a 24-well culture. Scramble-BV2 cells and TNFR2KD-BV2 cells were pretreated with 10 ng/mL of LPS for 24 h, and then re-seeded at the density of 1 × 10^4^ cells in C6 cell culture. The cultures were fixed by 4% paraformaldehyde at 24 h after co-culture with C6 cells and then incubated with 0.1% Triton X-100 for 20 min. The cultures were incubated with biotinylated isolectin B4 (IB4; Vector Laboratories, Burlingame, CA, USA) overnight at 4 °C, and reacted with Vectastain ABC kit (Vector Laboratories) for 1 h at room temperature. The staining was developed in the solution containing chromogen, 3,3′diaminobenzidine tetrahydrochloride (DAB) chromogen (Sigma, Saint Louis, MO, USA). The cultures were counterstained with hematoxylin and dehydrated in 95 and 100% alcohol solution.

Cell invasion analysis. Scramble-BV2 and *TNFR2*KD-BV2 cells were treated with 10 ng/mL of LPS for 24 h. The cells were replated at the density of 1 × 10^4^ cells/well onto PDL-pre-coated trans-well inserts with 8.0 μm pore size (Millipore, Burlington, MA, USA), and then co-cultured with C6 cells (3 × 10^4^ cells/well) for 6 h. The inserts were then removed and fixed in 4% paraformaldehyde for 10 min, followed by staining with 0.05% crystal violet in PBS for 10 min [25]. The upper surfaces of the trans-well inserts were carefully cleansed with cotton swabs. Similar to the procedure as previously described, BV2 cell migration was quantified by counting the number of cells that migrated through the membrane to the other side. BV2 cell invasion was determined by counting the cells on another side of the trans-well inserts from 5 randomly selected fields under a microscope. The results are represented as the average number of migrated cells per field.

Animal experiments. The C57BL/6 mice were obtained from Animal Center of National Cheng Kung University, Tainan, Taiwan. *TNFR2*-deficient (*TNFR2*^−/−^) mice on C57BL/6 backgrounds were originally from The Jackson Laboratory [26] and kindly provided by Dr. Chao-Ching Huang, Graduate Institute of Clinical Medicine, NCKU. The heterozygous mice (*TNFR2*^+/−^) were generated from breeding wildtype (WT) and *TNFR2*^−/−^ C57BL/6 mice. *TNFR2*^+/−^ littermates were then intercrossed to generate the offspring of WT, *TNFR2*^+/−^, and homozygous (*TNFR2*^−/−^) mice. Genotyping of the mice was done by examining wildtype *TNFR2* alleles using genomic DNA from the tail that was prepared from *TNFR2*^+/−^ and *TNFR2*^−/−^ mice at the age of 3 weeks old, and amplified with the specific primers through PCR analysis (Figure S1B). The primer sequences are shown in Table 1.

Peripheral administration of LPS. Adult C57BL/6 mice (24–28 g) received intraperitoneally (i.p.) with LPS (E. coli serotype 055:B5; Sigma) at the amount of 0.5 mg/kg once a day for 7 days. The control group received with Vehicle (phosphate-buffered saline; PBS) via i.p. injection once a day for 7 days.

Intracerebral glioma cell implantation. C6 and GL261 cell implantation was followed by the method as previously described with little modification [27]. Adult mice (24–28 g) were anesthetized with pentothal (60 mg/kg) and placed in a stereotaxic frame (Stoelting Co., Wood Dale, IL, USA). After the head was shaved and disinfected, a midline incision was made using a scalpel blade and the underlying tissue was removed using blunt dissection. With a dentist’s drill fitted with a 0.6-mm-diameter carbide dental burr (ELA, Engelskirchen, Germany), we drilled a hole in the exposed skull (stereotactic coordinates: 1 mm posterior to bregma and 1 mm to the right of sagittal suture). A Hamilton syringe with a 25-gauge needle was positioned on top of the hole, inserted into the brain, and advanced to the depth of 1.5 mm. The fluid (2 μL) containing 1 × 10^5^ of glioma cells was slowly injected into the cortical area just above the corpus callosum of mice. After each injection, the needle was maintained in the brain for an additional 2 min to reduce the possibility of leakage of the injected fluid from the site. After 14 days post glioma cell implantation (dpi), the mice were sacrificed by the perfusion of normal saline and 4% paraformaldehyde.

Animal perfusion. Animals were anesthetized via i.p. injection of pentothal (120 mg/kg), and perfused by DEPC-treated normal saline for RNA isolation. For cryoprotection, mice were perfused by normal saline and then 4% paraformaldehyde. After post-fixation in 4% paraformaldehyde for 3 days, the fixed brain tissues were cryoprotected in 30% (*w/v*) sucrose in PBS for 3 days. The brains were embedded in Tissue Tek OCT (Electron Microscopy Sciences, Hatfield, PA, USA) and then sectioned at 20 μm thickness.

Animal care and surgical protocol were provided in accordance with Laboratory Animal Welfare Act, Guide for the Care and Use of Laboratory Animals approved by the Institutional Animal Care and Use Committee of National Cheng Kung University (approval number 101249 and 102079). All surgery was performed under pentothal anesthesia, and all efforts were made to minimize suffering.

Immunohistochemistry (IHC). The tissue sections were incubated in 0.3% H_2_O_2_ in methanol for 15 min and in 0.1% Triton X-100 for 30 min. Tissues were then incubated with anti-Ki67 antibody (1:200; Abcam, Cambridge, UK), anti-Iba1 (1:200; Wako Pure Chemical, Osaka, Japan), and anti-MCP-1 antibody (Santa Cruz Inc., Santa Cruz, CA, USA) overnight, and followed by the biotinylated secondary antibody for 1 h at room temperature. The immunostaining for Ki67, Iba1, or MCP-1 was visualized using the Vectastain ABC kit (Vector Laboratories) and DAB chromogen. Subsequently, tissue sections were counterstained with hematoxylin and dehydrated in different percentages of alcohol solution (60, 70, 95, and 100%) and xylene. The tissue slides were mounted with coverslips using Entellan (Merck, Kenilworth, NJ, USA).

Double immunofluorescence. Cryostat tissue sections were permeabilized with 0.1% Triton X-100 for 30 min and incubated overnight with anti-MCP-1 (1:100) at 4 °C. On the following day, the antibody was washed off, and the sections were incubated with the appropriate secondary antibodies (1:200) for 1 h and Cy3-labeled avidin (1:200; Vector Laboratories) for 45 min. Subsequently, anti-Iba1 (1:200) antibody was applied at room temperature for 2.5 h. The tissue sections were then incubated with the appropriate secondary antibodies (1:200) for 1 h and then followed by FITC-labeled avidin D (1:500; Vector Laboratories) for 45 min. After being washed, the sections were counterstained with DAPI and mounted with coverslips in Permaflour. The results were analyzed under Nikon E800 fluorescence microscope.

Quantification of microglia in brain tissues Iba1^+^- or CD11b^+^-cells in brain tissue sections were manually quantified in the different brain regions from four series of tissue sections per animal using a phase-contrast microscope under 10 × objectives. Only cells with visible soma were counted using NIH ImagingJ. The values were represented as the average Iba1^+^- or CD11b^+^-cell number per field over.

Quantitative real-time PCR (Q-PCR)**.** Total RNA was isolated from the different brain regions using TRIzol^TM^ reagent (Invitrogen). Total RNA isolation and Q-PCR were performed as described previously [28]. One microgram of total RNA was then used for reverse transcription reaction using MMLV-reverse transcriptase, and Q-PCR was performed as previously described [28]. Q-PCR assay for the expression of mouse TNFα, TNFR1, or TNFR2 mRNA was accessed with a Roche LightCycler^TM^ using LightCycler FastStart DNA Master SYBR Green I kit (Roche Diagnostics Corporation, Mannheim, Germany). The results were normalized to *cyclophilin A* (*CyPA*) and expressed as a ratio of *TNFα*, *TNFR1*, or *TNFR2* to the *CyPA* control. The specific primers were designed for *TNFα*, *TNFR1*, *TNFR2*, and *CyPA* using LightCycler Probe Design Software 2 (Roche Diagnostics) and synthesized by MWG Biotech AG (Ebersberg, Germany). For gel-based RT-PCR, the amplification reaction was repeated for 30 cycles, and the reactants were separated by 1.5% agarose gel electrophoresis and stained by ethidium bromide to visualize the PCR products. The primer sequences are listed in Table 1.

Quantification of glioma tumor size. The method was adapted from the report by Morrone et al. [29] and followed by the method described previously [26]. The volume (mm^3^) of the tumor was derived from the tumor area (mm^2^) × number of slices × thickness of slices (20 μm).

Statistical analysis. Data are expressed as mean ± SEM. Each experiment was repeated at least three times. Statistical significance of differences between the two groups of data (*p*-value < 0.05) was analyzed using one-way analysis of variance (ANOVA) or two-way ANOVA test with Tukey’s comparisons.

## 3. Results

### 3.1. Growth and Migration of Microglia Increased by TNFR2 Gene Knockdown

**Figure 1:**
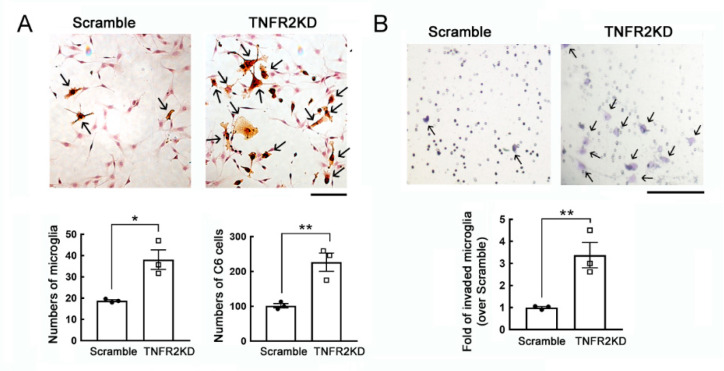
An increase in the growth and invasive ability of the LPS-primed mouse BV2 microglia cell line with TNFR2 gene knockdown in the co-culture with C6 glioma cells. (**A**). Scramble-BV2 cells and TNFR2KD-BV2 cells were treated with 10 ng/mL of LPS for 24 h, and then co-cultured with C6 glioma cells for another 24 h. The cultures were subjected to B4 isolectin (IB4) staining to identify BV2 cells. Scramble-BV2 cells or *TNFR2*KD-BV2 cells with IB4-positive staining show an active hypertrophic shape (arrows). The quantification of IB4^+^-BV2 cells and C6 glioma cells in the co-cultures were quantified under a microscope. (**B**). Scramble-BV2 cells and *TNFR2*KD-BV2 cells were seeded on the upper chamber compartment and primed with 10 ng/mL of LPS for 24 h. The cells were then co-cultured with C6 glioma cells replated onto the lower compartment for 6 h. The invasive *TNFR2*KD-BV2 cells (arrows) were fixed, stained with crystal violet, and quantified under a microscope. Data are presented as mean ± SEM. The experiments were repeated three times. Each dot (● or □) represents one-time experiment. Scale bar in A, 100 μm; in B, 50 μm. * *p* < 0.05, ** *p* < 0.05 versus Scramble.

To determine if glioma cells increased by co-culture with LPS-activated microglia with TNFR2 downregulation, we were first to conduct the in vitro study using a co-culture system of C6 glioma cells with a mouse BV2 cell line. Through lentivirus-mediated *TNFR2* shRNA approach, TNFR2 gene expression was significantly reduced in BV2 cells with no effect on the level of TNFR1 expression (Figure S1A). In the co-culture system, BV2 cells were pre-treated with LPS for 24 h and continued to co-culture with C6 glioma cells for another 24 h. The BV2 cells were recognized by IB4 staining in the co-culture system (Figure 1A, arrow). The results showed that BV2 cells were increased in C6 glioma co-cultures when compared to Scramble-BV2 cells (Figure 1A). Moreover, C6 glioma cells were increased in number when those cells co-cultured with *TNFR2*KD-BV2 (Figure 1A). In addition, the cell invasion of BV2 cells with *TNFR2*KD in the presence of LPS stimulation was greater than that of Scramble-BV2 cells (Figure 1B). The findings indicate that *TNFR2*KD promoted microglia invasive capability and proliferation, and increased glioma cell proliferation.

### 3.2. Enhanced Microglial Activation in TNFR2-Deficient Mouse Brain by Peripheral LPS Administration

**Figure 2:**
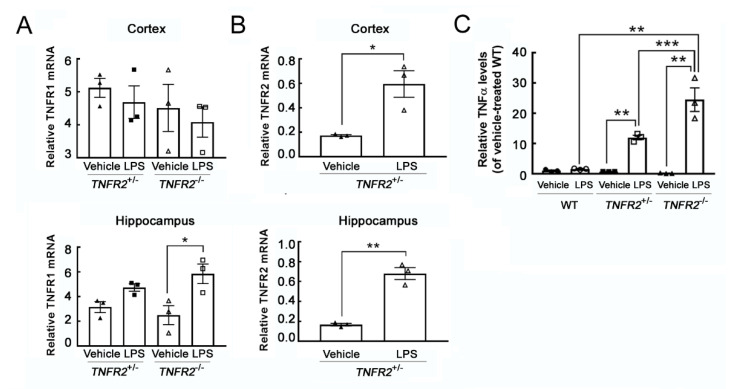
TNFR1 and TNFR2 mRNA expression in the brain regions of *TNFR2*^+/−^ and *TNFR2*^−/−^ mice receiving peripheral LPS injections. (**A**) Total RNA was isolated from the cortex and hippocampus of *TNFR2*^+/−^ and *TNFR2*^−/−^ mice after the peripheral injection with Vehicle or LPS (0.5 mg/kg/day) for 7 days. The samples were subjected to QPCR analysis for the measurement of TNFR1. (**B**) Total RNA was isolated from cortex and hippocampus of *TNFR2*^+/−^ mice receiving a daily peripheral injection with Vehicle or LPS (0.5 mg/kg/day) for 7 days, and then subjected to QPCR for the measurement of TNFR2 mRNA expression. (**C**) The cortical tissues were dissected from wildtype (WT), *TNFR2*^+/−^ and *TNFR2*^−/−^ mice after a 7-day injection with Vehicle or LPS (0.5 mg/kg/day). Total RNA was isolated and then subjected to QPCR for the measurement of TNFα mRNA expression. Data are presented as mean ± SEM (*n* = 3 animals per group). One dot indicated by the different symbol (◯, ●, □, ■, ▲, or △) represents an animal. * *p* < 0.05, ** *p* < 0.01, *** *p* < 0.001.

To investigate if peripheral inflammation induced by LPS was able to increase microglia activation in TNFR2 deficient mice, we used TNFR2 heterozygous mice (TNFR2^+/−^) and *TNFR2* knockout mice (*TNFR2^−/−^*). Through the genotyping by PCR to examine TNFR2 gene ablation in *TNFR2^−/−^* mice, TNFR2 gene was only observed in *TNFR2^+/−^* mice (Figure S1B), whereas neomycin resistance gene which was able to be detected in *TNFR2^+/−^* and *TNFR2^−/−^* mice. The results from further experiments by daily administration of LPS at 0.5 mg/kg for 7 days indicated that TNFR1 gene expression in the cortex was not changed by LPS administration, although TNFR1 mRNA levels were increased in the hippocampus of LPS-pretreated *TNFR2^−/−^* mice (Figure 2A). Yet, TNFR2 gene expression was increased by LPS administration in the cortical and hippocampal regions of *TNFR2^+/−^* mice (Figure 2B). To determine if inflammation was induced in the cortex of LPS-treated mice, the expression of proinflammatory cytokine TNF-α in the cortex was examined. TNFα mRNA levels were increased in the cortical regions of LPS-treated *TNFR2^−^/^−^* mice than that detected in LPS-treated WT or LPS-treated *TNFR2^+/−^* (Figure 2C and Figure S1C).

**Figure 3:**
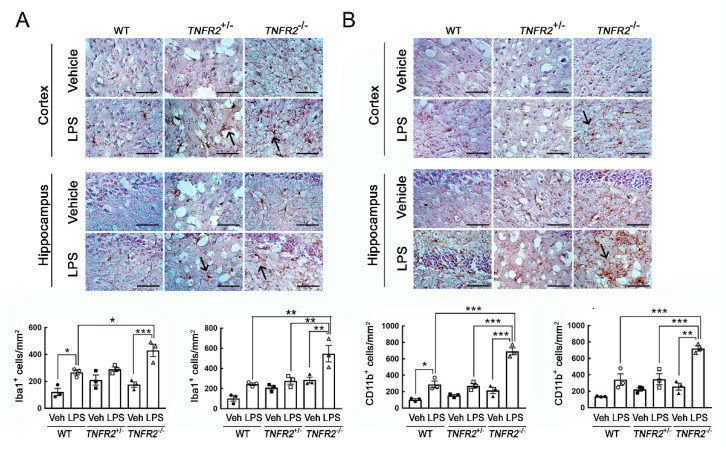
An increase in Iba1^+^-microglia in the cortex and hippocampus of *TNFR2^−/−^* mice receiving daily peripheral LPS injection for 7 days. Brains were removed from WT, *TNFR2^+/−^*, and *TNFR2^−/−^* mice after a 7-day injection with Vehicle (Veh) or LPS (0.5 mg/kg/day). The brain tissue sections were then subjected to immunohistochemistry for Iba1 (**A**) or CD11b (**B**). Iba1^+^-microglia and CD11b^+^-microglia were quantified using NIH ImagingJ as described in the Materials and Methods. Arrows indicate Iba1^+^-cells (**A**) or CD11b^+^-cells (**B**) with the complex processes. Data shown in A and B are presented as mean ± SEM (*n* = 3 animals per group). One dot indicated by the different symbol (O, ●, □, ■, ▲, or △) represents an animal. * *p* < 0.05, ** *p* < 0.01, *** *p* < 0.001. Scale bar, 100 μm.

Examination of microglia identified by Iba1 or CD11b immunostaining showed that Iba1^+^-microglia or CD11b^+^-microglia in the cortex and hippocampus were significantly increased in LPS-*TNFR2*^-/-^ mice when compared to that observed in WT and *TNFR2*^+/−^ mice (Figure 3). Note that LPS administration also increased the amount of Iba1^+^-microglia or CD11b^+^-microglia in the cortex and hippocampus of WT and *TNFR2*^+/−^ mice to some extent (Figure 3). The morphological observations also indicated that Iba1^+^-microglia in LPS-treated animal groups displayed a shape with thickening and branching proximal processes (Figure 3A, arrows). Microglia with intense CD11b immunoreactivity was observed in the cortex and hippocampus of *TNFR2*^+/−^ mice (Figure 3B, arrows). These results indicate that LPS-induced microglia activation and inflammation in the cortex and hippocampus was enhanced in mice with *TNFR2* deficiency.

### 3.3. Progressive Growth of Rodent Glioma Cells in TNFR2 Deficient Mice Receiving Peripheral LPS Administration

**Figure 4:**
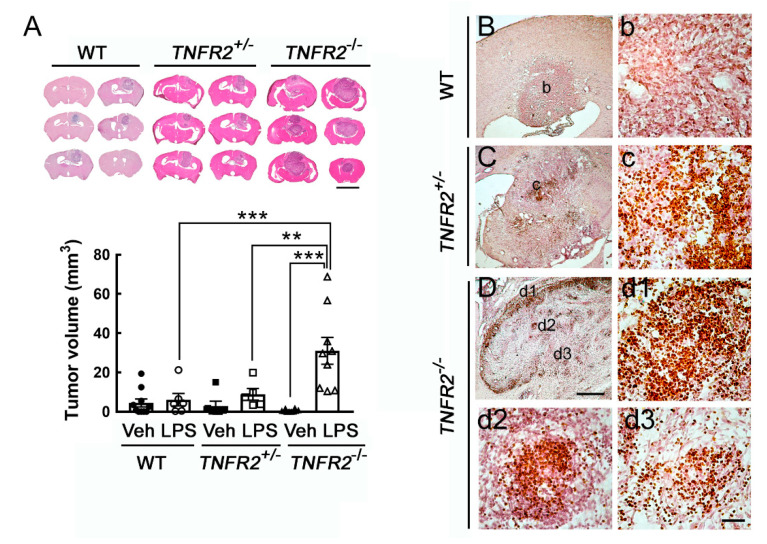
Enhanced glioma growth in *TNFR2*^−/−^ mice receiving repeated peripheral LPS injection for 7 days. (**A**). WT, *TNFR2*^+/−^, and *TNFR2*^−/−^ mice were treated with Vehicle (Veh) or LPS via i.p. injection (0.5 mg/kg/day) for 7 days, and then received intracerebral administration of C6 glioma (1 × 10^6^ cells/animal) into their cortex. Animals were sacrificed on day 14 after C6 implantation, and brains were removed for tissue sectioning and H/E staining (upper panel). The tumor volume of glioma-bearing animals was quantified. Data are presented as mean ± SEM (WT-Veh = 10, WT-LPS = 6; *TNFR2*^+/−^-Veh = 6, *TNFR2*^+/−^-LPS = 5; *TNFR2*^−/−^-Veh = 5, *TNFR2^−/−^*-LPS = 9). Each symbol (●, O, ■, □, ▲, or △) represents an animal. ** *p* < 0.01, *** *p* < 0.001. (**B**–**D**). Brain tissue sections collected from glioma bearing WT (**B**,**b**), *TNFR2*^+/−^ (**C**,**c**), and *TNFR2*^−/−^ (**D**,**d1**–**d3**) mice with a 7-day LPS injection were subjected to Ki67 immunostaining. The images with a higher magnification (**b**,**c**,**d1**–**d3**) were taken from the areas indicated in (**B**–**D**). Scale bar in A, 5 mm; in (**B**–**D**), 500 μm; in (**b**–**d**), 50 μm.

**Figure 5:**
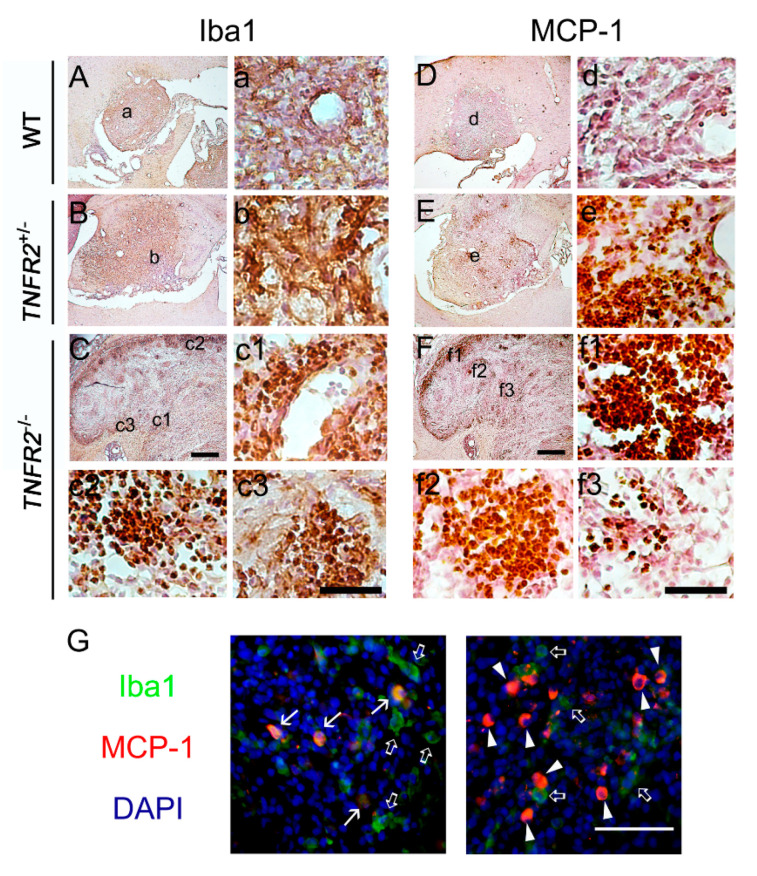
Accumulation of Iba1^+^ microglia and MCP-1^+^ cells in *TNFR2*^−/−^ mice receiving repeated peripheral LPS injection for 7 days. The brain tissue sections collected from glioma bearing WT (**A**,**D**), *TNFR2*^+/−^(**B**,**E**), and *TNFR2*^−/−^ (**C**,**F**) mice with repeated LPS treatment for 7 days were subjected to Iba1 (**A**–**C**) or MCP-1 (**D**–**F**) immunostaining. The images with a higher magnification (**a**–**f3**) were taken from the areas indicated in (**A**–**F**). In addition, double immunofluorescence for Iba1 (green)/MIP-1α (red) was performed to examine Iba1^+^-microglia expressing MCP-1 (arrows) in the glioma of *TNFR2*^−/−^ mice receiving a 7-day injection with LPS (0.5 mg/kg/day). Open arrows and arrowheads indicate Iba1^+^/MCP-1^−^ cells and Iba1^−^/MCP-1^+^ cells, respectively. The tissues were also subjected to DAPI nuclear counterstaining (blue). Scale bar in (**A**–**F**), 500 μm; in (**a**–**f**) and (**G**), 50 μm.

We noticed no biostatistical difference in tumor size among the three animal groups without peripheral LPS challenges, although the tumor volume in *TNFR2*^−/−^ mice displayed an increased trend when compared to that in WT and *TNFR2*^+/−^ mice. Yet, these animals survived for the entire experiment period. To examine tumor growth in the inflamed brain regions, we performed peripheral LPS administration before intracerebral implantation of C6 glioma cells into the animal groups (WT, *TNFR2*^+/−^, and *TNFR2*^−/−^). We noticed that the tumor size was shown as 2.5 ± 2.0 mm^3^ in WT mice, 2.9 ± 2.4 mm^3^ in *TNFR2*^+/−^ mice, and 6.1 ± 3.4 mm^3^ in *TNFR2*^−/−^ mice without peripheral LPS challenges. In the animal groups (WT, *TNFR2*^+/−^, *TNFR2*^−/−^ mice) daily pre-treated with 0.5 mg/kg of LPS for 7 days before intracerebral implantation of C6 glioma cells, the averaged mortality rate at 14 dpi from these animal groups was about 80%. Thus, the volume of C6-formed glioma in rats that survived at 14 dpi was measured. As shown in Figure 4A, the tumor formed by C6 glioma cells was enhanced in *TNFR2*^−/−^ mice treated LPS, when compared to those examined in LPS-treated WT or LPS-treated *TNFR2*^+/−^ mice. The results were in accordance with the observations by Ki67 immunohistological staining showing that the clusters of Ki67^+^-cells were widespread in the tumor of LPS-treated *TNFR2*^−/−^ mice at 14 dpi (Figure 4D,d1–d3). Moreover, Iba1^+^-microglia were accumulated in the tumor site of LPS-treated *TNFR2*^−/−^ mice (Figure 5C,c1–c3). Yet, Ki67^+^-cell clusters and were scattered in the tumor of LPS-pretreated *TNFR2*^+/−^ mice (Figure 4C,c). However, Ki67^+^-cells were rarely observed in the tumor formed in the cortex of LPS-treated WT mice (Figure 4B,b). Iba1^+^-microglia were able to be observed in LPS-treated WT (Figure 5A,a) and LPS-treated *TNFR2*^+/−^ mice (Figure 5B,b). The results reveal that glioma progression might be exacerbated in LPS-treated mice with loss of TNFR2 function.

In our previous study, we have observed infiltrating macrophages/microglia in glioma expressed MCP-1 [27]. Similar to the observations showing the distribution of Iba1^+^ microglia, in this study, MCP-1^+^ cells were abundantly amassed in the tumor of LPS-treated *TNFR2*^−/−^ mice (Figure 5F,f1–f3). However, MCP^+^-cells were also found in LPS-treated *TNFR2*^+/−^ mice (Figure 5E,e). Note that fewer MCP-1^+^ cells were observed in the tumor of LPS-treated WT mice (Figure 5D,d). Through double immunofluorescence, we observed that MCP-1 expression was co-localized to Iba1^+^-microglia/macrophages in the tumor of LPS-treated *TNFR2*^−/−^ mice (Figure 5G, arrows). Yet, the population of Iba1^+^-microglia/macrophages without MCP-1 immunoreactivity were found in the tumor (Figure 5G, open arrows). MCP-1^+^ cells without Iba1 immunoreactivity were largely accumulated in the tumor of LPS-treated *TNFR2*^−/−^ mice (Figure 5G, arrowheads). The results indicate that not only infiltrating microglia/macrophages, but also tumor cells, might serve as MCP-1 producing cell sources in the glioma to facilitate the infiltration of microglia/macrophages.

### 3.4. Progressive Growth of Mouse GL261 Glioma Cells in TNFR2 Deficient Mice Receiving LPS Pretreatment

**Figure 6:**
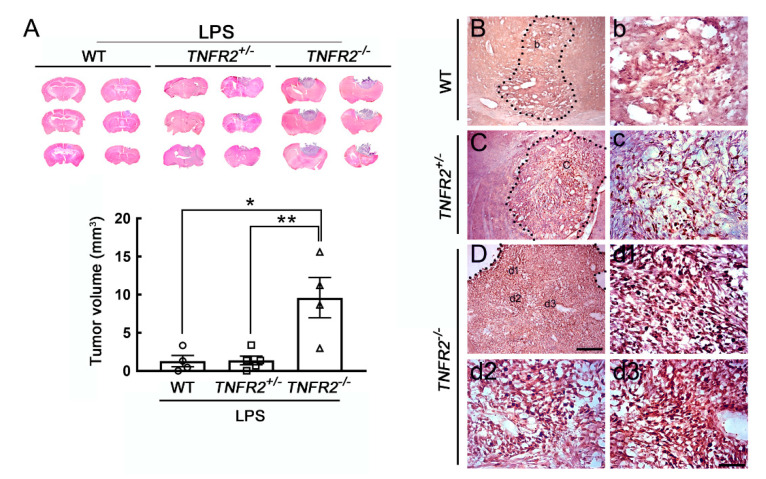
Enhanced tumor growth of mouse GL261 glioma cells in LPS-primed *TNFR2* deficient mice. (**A**) WT, *TNR2*^+/−^ or *TNR2*^−/−^ mice were intraperitoneally challenged with LPS at 0.5 mg/kg daily for 7 days, and then the three animal groups were treated with stereotactic intracerebral implantation of mouse GL261 glioma cells. The brain was removed at 14 dpi and sectioned into 20 μm thick cryosections of the three animal groups for H/E staining (upper panel). The bottom panel shows the quantitative data for the tumor volume (mm^3^), which are presented as mean ± SEM (WT-LPS mice = 4, *TNFR2*^+/−^-LPS mice = 5, *TNFR2*^−/−^-LPS mice = 4). Each symbol (O, □, or △) represents an animal. * *p* < 0.05, ** *p* < 0.01 compared to the WT-LPS mice. (**B**–**D**) The brain tissue sections collected from glioma-bearing WT (**B**,**b**), *TNFR2*^+/−^ (**C**,**c**), and *TNFR*2^−/−^ (**D**,**d1**–**d3**) mice with a 7-day LPS injection were subjected to Ki67 immunostaining. Scale bar in A, 5 mm; in (**B**–**D**), 200 μm; in (**b**–**d**), 50 μm.

Furthermore, we performed the intracerebral implantation of syngeneic mouse glioma cell line GL261 to verify the tumor-supporting role of activated microglia in glioma growth. Consistent with the observation in the model of C6 xenotransplantation (Figure 4), the tumor volume formed by GL261 cells in LPS-treated *TNFR2*^−/−^ mice was significantly greater than that observed in LPS-treated WT or *TNFR2*^+/−^ mice (Figure 6A). The tumor size in the three Vehicle-treated animal groups was approximately at the region of 0.77 ± 0.19 mm^3^. Yet, similar to the results shown in Figure 4B–D, Ki67^+^ cell clusters were abundantly found in the tumor in LPS-treated *TNFR2*^−/−^ mice at 14 dpi (Figure 6D,d1–d3), whereas the scattering of Ki67^+^ cells was observed in the tumor of LPS-treated *TNFR2*^+/−^ mice (Figure 6C,c). It was noted that rare Ki67^+^ cells existed in the tumor of LPS-treated WT mice (Figure 6B,b). Thus, these results further confirm that the expansion of glioma cells was enhanced in *TNFR2*-deficient mice with LPS treatment, which could be due to the inflammatory microenvironment induced by peripheral LPS challenge and loss of TNFR2 signaling.

## 4. Discussion

Clinical evidence that TNFR2 expression was lower in the monocytes derived from glioblastoma suggests that the defect of TNFR2 expression plays a critical factor in the low yields of mature dendritic cells in the peripheral blood of glioblastoma patients [23]. However, to date, poor evidence shows the link of reduced TNFR2 action with glioma progression. In the study, we present that peripheral LPS challenge significantly increased TNFα expression and Iba1^+^-/CD11b^+^-activated microglia in the cortical and hippocampal regions of *TNFR2*^−/−^ mice than those of WT or *TNFR2*^+/−^ mice. Moreover, in conjunction with the findings of aggressive glioma growth in the brain of LPS-primed *TNFR2*^−/−^ mice, our study demonstrates that activated microglia might promote glioma expansion in the tumor-initiating site.

The findings have shown that the highest amount of microglia was detected in the cortex of adult C57BL/6J mice after an intraperitoneal injection of 1 mg/kg of LPS [30]. The studies from others have reported that microglia activation was mediated by TNFα signaling after peripheral LPS injection [31,32]. Our present study shows that the upregulation of TNFR1 expression was only observed in the hippocampus of *TNFR2*^+/−^ and *TNFR2*^−/−^ mice after the repeated peripheral injection of LPS at 0.5 mg/kg for 7 days, indicating that peripheral injection with LPS at this dose was not enough to cause a change in TNFR1 expression globally in the brain of *TNFR2*^+/−^ and *TNFR2*^−/−^ mice. Yet, our results reveal that TNFα mRNA was expressed at a much higher level in the cortex of LPS-treated *TNFR2*^−/−^ mice than that of LPS-treated *TNFR2*^+/−^ group. The findings also provide evidence showing the activation of microglia significantly observed in the cortical region of *TNFR2*^−/−^ animal group by peripheral injections with LPS. Yet, we noticed that TNFα expression observed in the indicated brain regions was not altered at 7 days post daily administration of LPS in the WT group. The observations are very similar to the findings showing that the release of brain proinflammatory cytokines was significantly abolished by daily i.p. injections of 0.5 mg/kg LPS for 4 days [33]. This inhibition is referred as immune tolerance. Based on our results, perhaps, the regulatory role of TNFR2 signaling in immune tolerance can be further studied in the future.

The soluble TNFRs (sTNFRs) have been reported to serve as potent inhibitors of TNFα activity by neutralization of TNFα [34,35,36]. However, at low concentrations, sTNFR can stabilize trimeric TNF and expand its availability for binding to the membrane receptors, thus increasing TNF activity [37,38]. Since an increased level of soluble TNFR2 (sTNFR2) is detected in the serum of peripheral LPS-treated animals [39], the reduction or loss of TNFR2 function may increase TNFα bioactivity in *TNFR2*^−/−^ mice. An in vitro study carried out by others showed that activation of TNFR2 in LPS-treated microglia could promote the secretion of anti-inflammatory cytokines [40]. Alternatively, it has been reported that microglial activation can be stimulated by TNFα/TNFR1 signaling triggered by peripheral inflammation [30,32,41]. Moreover, genetic deletion of TNFR1 has been found to alleviate microglia activation in mice with overexpression of mutant amyloid precursor protein [42]. These studies support our observations that the deficiency of TNFR2 signaling enhanced brain inflammation under the influence of LPS-induced peripheral inflammation. It is also possible that microglial activation enhanced in LPS-treated mice with TNFR2 gene deficiency might be mediated by TNFα/TNFR1 action.

The amount of infiltrating microglia/macrophages is positively correlated to the higher grade of glioma [1,43]. The evidence from ex vivo experiments has shown that rat F98 or mouse GL261 glioma cells applied onto the brain slice can stimulate metalloprotease-2 activity derived from exogenous microglia, which in turn enhance glioma cell invasiveness [13]. The findings from Tsirka and his coworkers using in vitro coculture system have also demonstrated that activated microglia promote the growth of mouse glioma GL261 cells, whereas GL261 cells suppressed microglial phagocytic response [11]. Glioma growth can be reduced by local ablation of microglia/macrophages in CD11b-HSVTK transgenic animal models through a pharmacological approach [11]. Moreover, the activation of microglia/macrophages by Tuftsin (threonine-lysine-proline-arginine, TKPR), a potent stimulator, can increase the tumor size in glioma-bearing mice [11]. Despite that the application of CpG oligonucleotides (CpG ODN), an immunostimulant, has been reported to promote glioma apoptosis along with enhancement of microglial antigen-presenting capacity [44], the other findings have shown that the tumor size was enhanced in rat 9L gliosarcoma-bearing Fisher 344 rats receiving an intratumoral injection of CpG ODN [45]. Moreover, the immunosuppressant, cyclosporine A, exerts potent effects on the inhibition of glioma-associated microglia activity and on the reduction of microglia-induced glioma invasiveness [12]. These works demonstrate that activated microglia possess pro-tumorigenic activity and glioma-associated microglia/macrophages can be stimulated by tumor-secreted factors to promote glioma growth. Our in vitro observations that C6 glioma cell growth was enhanced by LPS-primed mouse microglia BV2 cells with TNFR2 gene knockdown (Supporting Information S1). Thus, our in vivo results that pretreatment with LPS enhanced glioma growth in *TNFR2*^−/−^ mice receiving LPS pretreatment might be partly due to activated microglia-created inflamed microenvironment.

MCP-1 expression has been reported to be related to the grade of malignancy and microglial infiltration in human gliomas [46,47]. Microglia can produce MCP-1 through TNFα/TNFR1 signaling triggered by peripheral inflammation [20,32,41], which, in turn, attracts the migration of microglia/macrophage into the tumor [48,49]. Similar to our previous findings in glioma-bearing rats [26], we have observed that MCP-1^+^/Iba1^+^ cells exist in the glioma of LPS-primed *TNFR2*^−/−^ mice. Yet, MCP-1^−^/Iba1^+^ cells are also observed in tumors of LPS-primed *TNFR2*^−/−^ mice. This demonstrates that infiltrating microglia/macrophages in the tumor are at different activation statuses [27]. Moreover, the findings have demonstrated that glioma secretes MCP-1 to induce the infiltration of microglia/macrophages [1,46,47,50], in this study, the massive amount of MCP-1^+^/Iba1^−^ cells might serve as a chemokine-producing source for the recruitment of Iba1^+^ microglia/macrophages in glioma. Alternatively, given the fact that endothelial cells are considered as the tumor-supporting cells via secreting growth factors to promote glioma cell proliferation and angiogenesis [51], it is important to uncover the dysregulation of TNFR2 signaling in the function of endothelial cells. In addition, it also remains to be studied if the lack of TNFR2 signaling in brain endothelial cells induces the dysfunction of blood–brain barrier and leads to the exaggeration of the immune cell infiltration and tumor progression.

## 5. Conclusions

Peripheral inflammatory challenge in combination with the lack of TNFR2 signaling enhances microglia activation and most likely creates an inflamed microenvironment in the brain, which could promote tumor progression in glioma-bearing animals. The study also provides important evidence to indicate the notion that the augmentation of microglia activation plays a regulatory role in the stimulation of glioma growth in the brain. These findings also suggest that TNFR2 function may prevent tumor expansion from the stimulation of the inflamed brain.

## Figures and Tables

**Table 1:** A list of primer sequences used for genotyping and Q-PCR analysis and sequences of shRNA against TNFR2.

Gene	Forward (5′->3′)	Reverse (5′->3′)
*TNFR2* allele (t)	AGCTCCAGGCACAAGGGCGGG	CCTCTCATGCTGTCCCGGAA
*neomycin-resistant gene* gene (neo^R^)	CACGGGTAGCCAACGCTATGTC	GCCCTGAATGAACTGCAGGACG
*CyPA*	CGTCTGCTTCGAGCTGTTTG	GTAAAATGCCCGCAAGTCAA
*TNFα*	ACCGTCAGCCGATTTGCTAT	CCGGACTCCGCAAAGTCTAA
*TNFR1*	GCTTGTGTCCCCAAGGAAAG	GTCCTGGGGGTTTGTGACAT
*TNFR2*	GTCGCGCTGGTCTTCGAACTG	GGTATACATGCTTGCCTCACAGTC
**shRNA**	**Sequence**	
sh-*ctrl* (Scramble)	AAAAGCTACACTATCGAGCAATTTTGGATCCAAAATTGCTCGATAGTGTAGC	
sh-*mTNFR2*-188	AAAAGCCAGATCTCACAGGAATATTGGATCCAATATTCCTGTGAGATCTGGC	
sh-*mTNFR2*-220	AAAAGCTCAGATGTGCTGTGCTATTGGATCCAATAGCACAGCACATCTGAGC	

## Data Availability

Data are available upon reasonable request.

## Supplementary Materials

The following are available online at https://www.mdpi.com/article/10.3390/life11090961/s1, Figure S1: Supplementary data show the efficiency of shRNA against TNFR2 expression in a microglia cell line, TNFR2 gene deficiency in TNFR2 knockout mice, and TNFα mRNA expression in the cerebral cortical tissues of animal groups receiving Vehicle or LPS; Figure S2: Original Western Blotting figure for Figure S1A; Figure S3: Original Western Blotting figure for Figure S1B.; Figure S4: Original Western Blotting figure for Figure S1C.
