# Supplementary material for: Enhanced Microglia Activation and Glioma Tumor Progression by Inflammagen Priming in Mice with Tumor Necrosis Factor Receptor Type 2 Deficiency
Source: Life (Basel). 2021 Sep 14;11(9):961. doi: 10.3390/life11090961 (PMC8465392; doi:10.3390/life11090961)
Supplement: Supplementary file 1 [file life-11-00961-s001.zip › life-1344784-supplementary.pdf]

life

MDPI

Article

Enhanced Microglia Activation and Glioma Tumor Progression by Inflammagen Priming in Mice with Tumor Necrosis Factor Receptor Type 2 Deficiency

Chih-Kai Liao [1,2,3,†], Kuan-Min Fang [1,†], Hui-Ting Huang [1], Wen-Ruei Chang [1], Chao-Chi Chuang [1] and Shun-Fen Tzeng [1,*]

[1] Institute of Life Sciences, College of Bioscience and Biotechnology, National Cheng Kung University, Tainan 70101, Taiwan; ckliao37@csmu.edu.tw (C.-K.L.); aleskuan@gmail.com (K.-M.F.); 10904022@gs.ncku.edu.tw (H.-T.H.); r9052000@hotmail.com (W.-R.C.); chaung.gi@gmail.com (C.-C.C.)
[2] Department of Anatomy, Faculty of Medicine, Chung Shang Medical University, Taichung 40241, Taiwan
[3] Department of Medical Education, Faculty of Medicine, Chung Shang Medical University, Taichung 40241, Taiwan
† Equal contribution
* Correspondence: stzeng@mail.ncku.edu.tw; Tel.: +886-6-2757575 (ext. 58129)

Supplementary

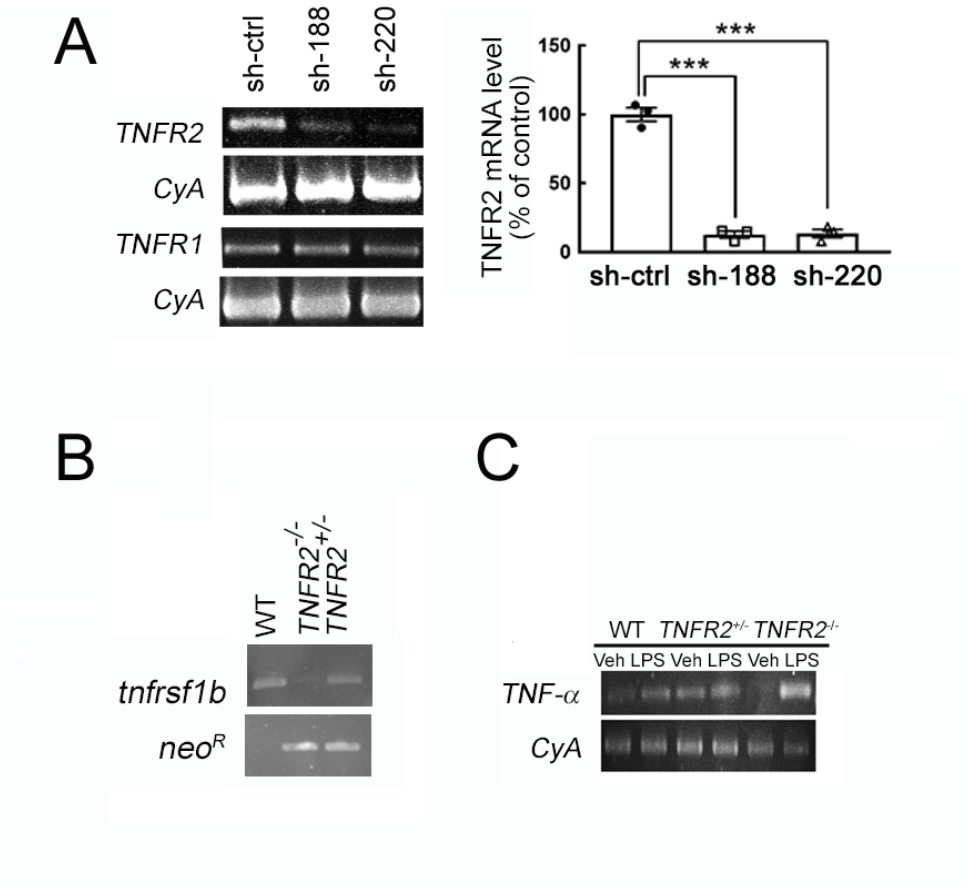

Figure S1. Supplementary data show the efficiency of shRNA against *TNFR2* expression in microglia cell line, TNFR2 gene deficiency in TNFR2 knockout mice, and TNFα mRNA expression in the cerebral cortical tissues of animal groups receiving vehicle or LPS. (A) BV2 cells were infected with lentivirus expressing control shRNA (sh-ctrl), or sh-*TNFR2* (sh-188 and sh-220). Total RNA was isolated from BV2 cells and then subjected to gel-based RT-PCR (left panel) and QPCR analysis (right panel) for the measurement of TNFR1 and TNFR2 mRNA levels. TNFR2 mRNA levels in BV2 cells were significantly reduced by lentivirus-mediated shRNA (sh-188 and sh-220) delivery. Note that TNFR1 mRNA expression in BV2 cells was not affected by lentivirus-mediated shRNA delivery for TNFR2 gene knockdown. Data are presented as mean ± SEM. The experiments were repeated three times. ****P* < 0.001 versus Scramble. (B) Genomic DNA was extracted from wildtype (WT), *TNFR2*$^{+/--}$, *TNFR2*$^{-/-}$ or, *TNFR2*$^{+/-}$ mouse tails, and then subjected to PCR for genotyping by using *tnfrsf1b* primers as described in Materials and Methods. The expression of *neomycin* resistance gene (*neo*^{R}) was detected in the tissues derived from TNFR2$^{-/-}$ or TNFR2$^{+/-}$ mice. (C) As described in Materials and Methods, the cerebral cortex was dissected from WT, *TNFR2*$^{+/-}$ and *TNFR*2$^{-/-}$ mice after a 7-day repeated injection with vehicle or LPS (0.5 mg/kg/day). Total RNA was isolated at Day 8, and then subjected to gel-based RT-PCR for the examination of TNFα mRNA levels and CyA (as an internal control).

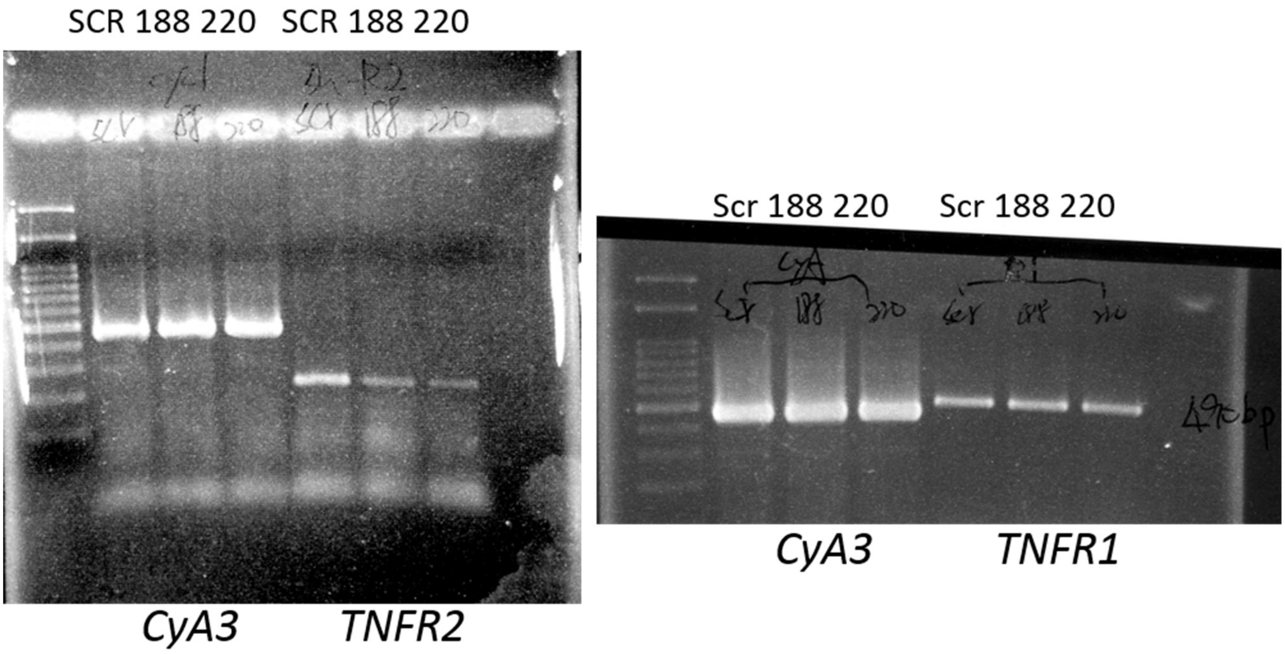

Figure S2. Original Western Blotting figure for Figure S1A.

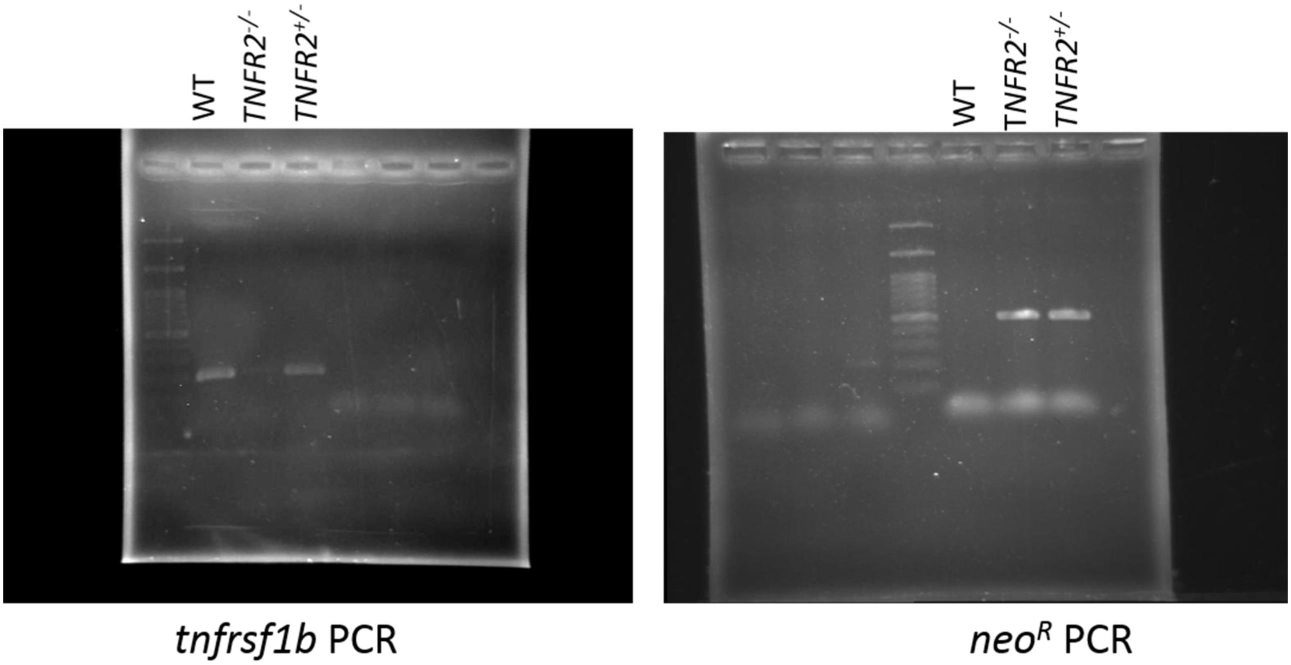

Figure S3. Original Western Blotting figure for Figure S1B.

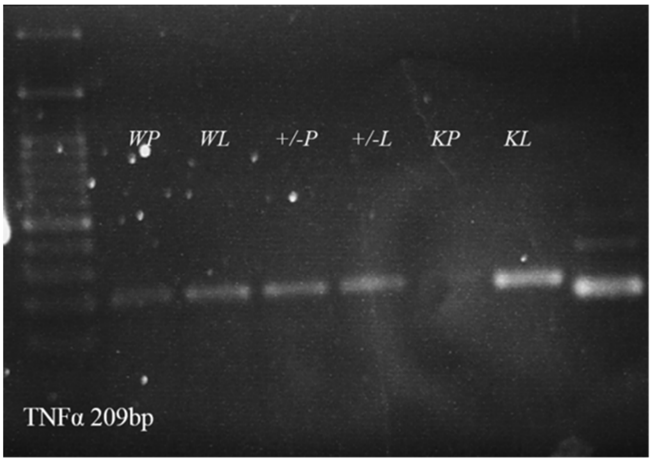

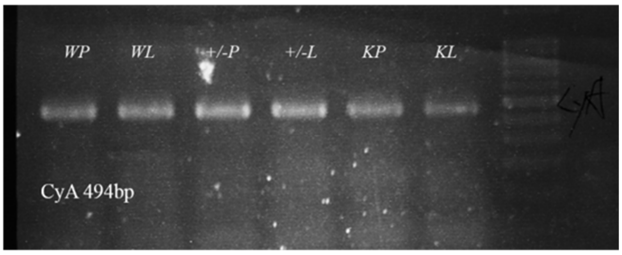

WP: WT/PBS; *WL*: WT/LPS
+/-P: *TNFR2*$^{+/-}$/PBS
+/-L: *TNFR2*$^{+/-}$/LPS
KP: *TNFR2*$^{-/-}$ /PBS
KL: *TNFR2*$^{-/-}$ /PBS

Figure S4. Original Western Blotting figure for Figure S1C.
